# Developing novel methods to image and visualize 3D genomes

**DOI:** 10.1007/s10565-018-9427-z

**Published:** 2018-03-26

**Authors:** Tszshan Ma, Long Chen, Maoxiang Shi, Jing Niu, Xu Zhang, Xusan Yang, Karl Zhanghao, Miaoyan Wang, Peng Xi, Dayong Jin, Michael Zhang, Juntao Gao

**Affiliations:** 1 School of Medicine, Tsinghua University, Beijing, 100084 China; 2 MOE Key Laboratory of Bioinformatics; Bioinformatics Division, Center for Synthetic & Systems Biology, BNRist; Department of Automation, Tsinghua University, Beijing, 100084 China; 3 Center for Synthetic & Systems Biology, Tsinghua University, Beijing, 100084 China; 4 Department of Basic Medical Sciences, School of Medicine, Tsinghua University, Beijing, 100084 China; 5 School of Life Sciences, Tsinghua University, Beijing, 100084 China; 6 Department of Automation, Tsinghua University, Beijing, 100084 China; 7 Department of Biomedical Engineering, College of Engineering, Peking University, Beijing, 100871 China; 8 Institute for Biomedical Materials and Devices (IBMD), Faculty of Science, University of Technology Sydney, Sydney, NSW 2007 Australia; 9 Department of Biological Sciences, Center for Systems Biology, the University of Texas at Dallas, Richardson, TX 75080-3021 USA

**Keywords:** 3D genomes, Chromatins, FISH method

## Abstract

To investigate three-dimensional (3D) genome organization in prokaryotic and eukaryotic cells, three main strategies are employed, namely nuclear proximity ligation-based methods, imaging tools (such as fluorescence in situ hybridization (FISH) and its derivatives), and computational/visualization methods. Proximity ligation-based methods are based on digestion and re-ligation of physically proximal cross-linked chromatin fragments accompanied by massively parallel DNA sequencing to measure the relative spatial proximity between genomic loci. Imaging tools enable direct visualization and quantification of spatial distances between genomic loci, and advanced implementation of (super-resolution) microscopy helps to significantly improve the resolution of images. Computational methods are used to map global 3D genome structures at various scales driven by experimental data, and visualization methods are used to visualize genome 3D structures in virtual 3D space-based on algorithms. In this review, we focus on the introduction of novel imaging and visualization methods to study 3D genomes. First, we introduce the progress made recently in 3D genome imaging in both fixed cell and live cells based on long-probe labeling, short-probe labeling, RNA FISH, and the CRISPR system. As the fluorescence-capturing capability of a particular microscope is very important for the sensitivity of bioimaging experiments, we also introduce two novel super-resolution microscopy methods, SDOM and low-power super-resolution STED, which have potential for time-lapse super-resolution live-cell imaging of chromatin. Finally, we review some software tools developed recently to visualize proximity ligation-based data. The imaging and visualization methods are complementary to each other, and all three strategies are not mutually exclusive. These methods provide powerful tools to explore the mechanisms of gene regulation and transcription in cell nuclei.

## Introduction

Spatial organization of chromatins plays a very important role in replication, repair, and transcriptional activity regulation (Cremer and Cremer 2001). There are three main strategies to study chromatin interactions in 3D genomes: high-throughput nucleus proximity ligation-based methods, imaging tools, and computational/visualization methods (Dekker 2016; Gao et al. 2016; Yu and Ren 2017). The first group of strategies covers the family of chromosome conformation capture (3C)-based methods, which involve digestion and re-ligation of physically proximal cross-linked chromatin fragments, accompanied by massively parallel DNA sequencing to measure the relative spatial proximity between genomic loci (de Wit and de Laat 2012; Dekker 2016; Diament and Tuller 2017; Mifsud et al. 2015; Rao et al. 2014). For example, Hi-C, one of the 3C-based methods, interrogates all possible cross-linked contacts at acceptable resolutions within the whole genome. Imaging tools enable direct visualization and quantification of spatial distances between genomic loci, and advanced implementation of (super-resolution) microscopy has helped significantly improve the resolution of images. Among the third group of strategies, computational methods are used to map global 3D genome structures at various scales driven by experimental data (such as 3C-coupled sequencing data) or by basic assumption of physical properties (such as polymer models of chromosomal regions; see review (Fudenberg and Mirny 2012; Varoquaux et al. 2014)), while visualization methods are used to visualize these data in virtual 3D space with the help of different algorithms (Gao et al. 2016).

In this review, we will mainly focus on the recent progress of some novel bioimaging methods and computational visualization techniques that are important for the second and third strategies mentioned above. We will first introduce imaging methods used to label and image genomic data in fixed cells, such as in situ hybridization (ISH) methods based on long fluorescent probes, short oligo probes, and molecular beacons. Next, we will present the methods used for imaging genomic loci in live cells. Because the fluorescence-capturing capability of a particular microscope is very important for the sensitivity of bioimaging experiments, we next provide a short introduction to the two novel super-resolution microscopy methods developed by us recently, polarized super-resolution microscopy (PSRM) and low-power super-resolution stimulated emission depletion (STED) microscopy. Then, we review the development of some visualization tools used for 3D genome study, with a focus on the two viewers developed recently: Web3DMol and HiC-3DViewer. We conclude with a discussion and a short outlook on future development, including how to integrate these strategies together.

## Imaging 3D genomes in fixed cells

### Imaging genomic loci with long probes

Before the advent of molecular techniques such as 3C (chromosome conformation capture) (Dekker et al. 2002) and their high-throughput derivatives, the predominant method for studying nuclear organization and chromatin conformation was based on DNA labeling coupled with microscopic observation. Methods that detect DNA without sequence specificity, such as the use of the general fluorescent DNA stain DAPI (Kapuscinski 1995) and the incorporation of fluorescent dNTPs, allowed us to catch a glimpse of chromatin DNA packaging and morphologies during various cell stages, including mitosis and DNA replication. Developing DNA sequence-specific labeling methods is required to understand any potential relationship between DNA sequence information (such as that of genes, regulatory elements) and their positioning within the nucleus. Fluorescence in situ hybridization (FISH), the most popular technique among numerous DNA labeling methods, detects specific DNA sequence based on the complementary base pairing between target DNA and probes. Since it was first invented in the 1980s (Langer-Safer et al. 1982), the FISH method has been modified to improve its sensitivity, specificity, and resolution power to cater to the needs of chromatin organization research at different levels accompanied by advancement in fluorescence microscopy technology and genomics and bioinformatics knowledge (Giorgetti and Heard 2016). The sensitivity of FISH experiments depends on the fluorescence signal intensity of the probes, the signal-to-noise ratio between the targets and background, and the labeling efficiency, as well as the fluorescence-capturing capability of a particular microscope.

**Fig. 1:**
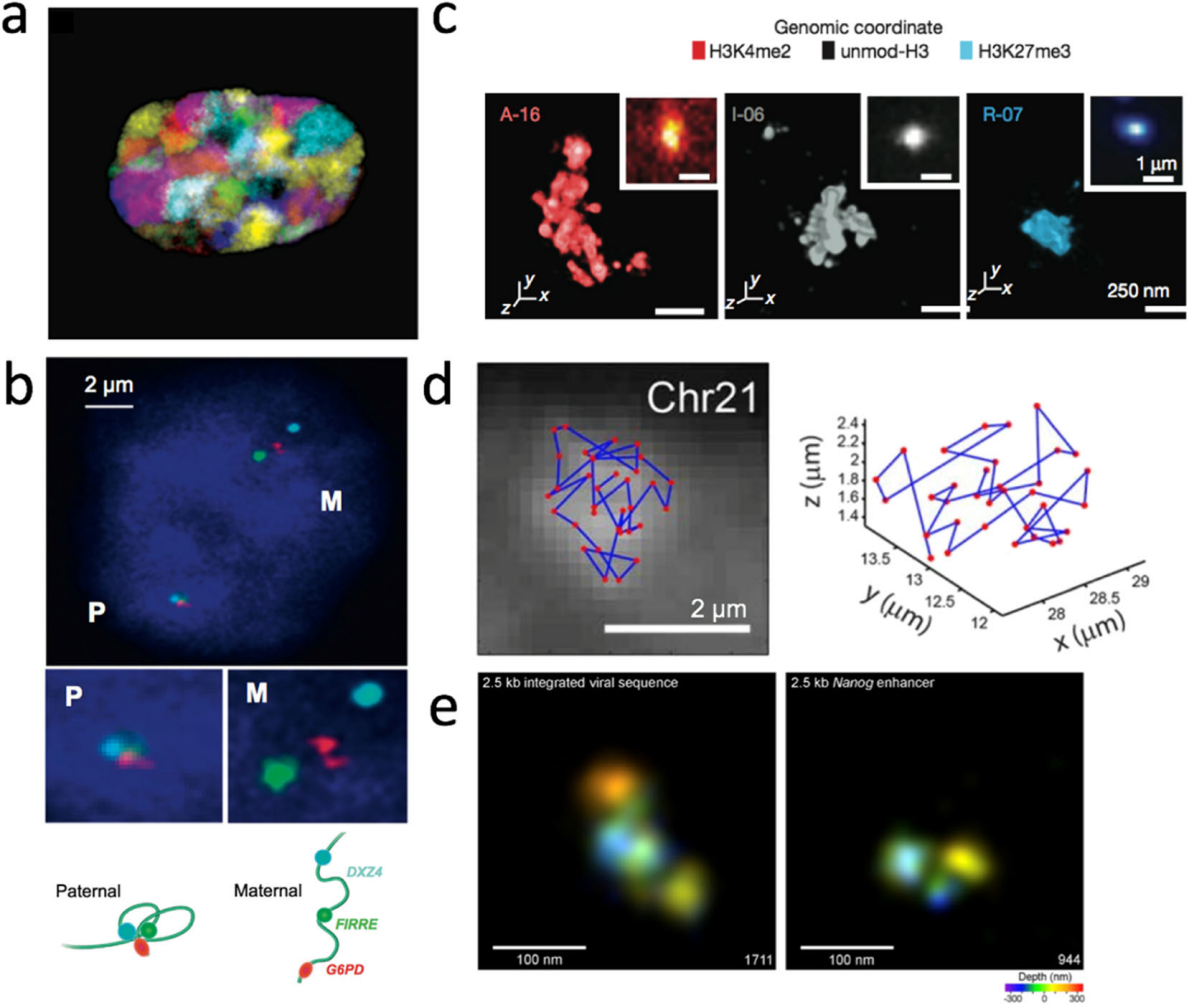
3D genome visualization based on DNA-FISH methods. **a** False color representation of the 24 differently labeled chromosome types (1–22, *X* and *Y*) with DNA-FISH demonstrating how each chromosome accommodates its respective chromosome territory (image modified from (Bolzer et al. 2005). **b** Three-color DNA-FISH validation of the Haplotype-specific super-long interactions mediated by CTCF connecting three loci: DXZ4, FIRRE, G6PD in ChrX (top). Loci in the paternal-origin chromosome showed specific localization (lower left) which is absent in the maternal-origin chromosome (lower right) (image modified from Tang et al. 2015). **c** 3D-STORM images of three distinct epigenetic domains (left: active, middle: inactive, right: repressed) labeled by “Oligopaint” FISH probes with photoswitchable dye Alexa-647, shown with their corresponding conventional images in the inset (image modified from Boettiger et al. 2016). **d** Mapping the spatial organization of the central 100-kb regions of 34 TADs in Chr21. The position of each TAD is plotted as red dot in the microscopy image (left) and in 3D (right) (image modified from Wang et al. 2016b). **e** 3D-STORM images of a 2.5-kb exogenous viral sequence integrated in the cell (left) and an endogenous sequence (right) labeled by MB probes with the photoswitchable dye Alexa 647 (image modified from Ni et al. 2017).

The size of the fluorescent probe and its target region are directly proportional to the strength of the signals. The long probes library generated from nick translation (Pinkel et al. 1988) or degenerate oligonucleotide primes PCR (DOP-PCR) (Telenius et al. 1992a) using cosmids, fosmids, ranges of artificial chromosomes (BACs, YACs and PACs), and flow-sorted chromosomes (Telenius et al. 1992b) can be employed to paint target genome loci ranging from a few hundred kilobases (kb) to the whole chromosome, with a probe resolution larger than 25 kb, depending on the choice of template for probe generation. Biological findings regarding the chromatin architecture at the chromosome level, such as visualizing chromatin territories and interchromatin space (Bolzer et al. 2005; Cremer and Cremer 2001) and various fascinating examples of long-range genomic interactions with important biological implications in cell differentiation, paternal-maternal specificity, etc. (Hogan et al. 2015; Tang et al. 2015) have been illustrated by long-probe FISH with conventional fluorescence microscopy (Fig. 1a, b). However, the genomic sequence resolution, that is, the ability to distinguish two separate loci along a chromosome, is compromised by the size of long probes. As one locus labeled with long probes would be of a size between 40 kb and more than 200 kb, two loci separated by genomic distance smaller than 100 kb cannot be resolved or measured very efficiently; biologically meaningful chromatin interactions can range from tens of kb to 1 Mb (mega base), or the loci of interest might only be a few kilobases in size (e.g., regulatory elements such as promoters and enhancers) (Boettiger et al. 2016; Williamson et al. 2014). Sequence-specific probes targeting a smaller sized genome region (< 10 kb) are needed to label the target with higher precision and to study those interactions within 100-kb genome domains. It is also important to note that the spatial resolution power of conventional light microscopy is limited to 200 nm in the lateral plane (*x*-*y* plane) and 500 nm along the axial direction (*z*-axis). Hence, the incorporation of super-resolution microscopy (details discussed in the “Novel super-resolution microscopy” section), which decreases the diffraction limit of light to the scale of tens of nanometers, is essential for viewing short-range chromatin interactions because a 100-kb-long chromatin would be at the scale of tens of nanometers within the nucleus depending on its epigenetic states (Boettiger et al. 2016; Williamson et al. 2014).

### Imaging genomic loci with short probes

Short probe development and utilization in DNA labeling depends greatly on the various approaches in synthesizing fluorophore-tagged oligonucleotides (oligos). Because it is expensive to custom-synthesize hundreds of fluorescent oligos separately to label one specific loci, they were more frequently used to label specific genomic regions with highly repetitive sequence motifs such as in telomeres and centromeres (Matera and Ward 1992). Different single-strand oligo probe synthesis strategies with lower production costs and simplified procedures were proposed to promote the use of these probes in FISH experiments (Beliveau et al. 2012; Boyle et al. 2011). With such PCR-based preparation, the target size of the probe could be adjusted freely as long as the total fluorescence intensity generated by the probe pool could be distinguished from background under microscopy. While Boyle et al. could generate usable oligo probes to label genomic loci as short as 15 kb in length with a hybridization efficiency of ~ 70%, they could not reliably probe targets of a smaller size under confocal microscopy (Boyle et al. 2011). To improve the hybridization efficiency of small genomic loci, Beliveau et al. augmented the fluorescence signal intensity of oligo probes with secondary fluorophore-tagged oligos (Beliveau et al. 2015) and coupled it with stochastic optical reconstruction microscopy (STORM) (Rust et al. 2006) to attain super-resolution imaging. These modified “Oligopaint” probes allowed detection of loci as small as 5 kb (Beliveau et al. 2015) and accurate measurement of the labeled chromatin volume in genome regions with different epigenetic states (Fig. 1c), providing the first direct evidence of how the epigenetic state leads to distinct chromatin folding (Boettiger et al. 2016). The robustness of the “Oligopaint” method further allowed the sequential labeling and detection of multiple loci in single cells and the measurement of the inter-locus distance to generate a 3D loci-inter-distance-relation map in sequence resolution comparable to that in Hi-C (Fig. 1d) (Wang et al. 2016b). As an alternative to intensify signals by using a secondary intensifying probe, Ni et al. designed and synthesized oligo-probes in the form of molecular beacons tagged with fluorophores and quenchers on each end, termed MB-FISH (Molecular Beacon-FISH), to label non-repetitive DNA (Ni et al. 2017). The self-complementarity of MB probes reduced false positive signals introduced by leftover non-specific binding probes and hence increased the signal-to-noise ratio, allowing the detection and 3D depiction of specific DNA loci as short as 2.5 kb in fine detail using 3D-STORM (Fig. 1e) (Ni et al. 2017). With high labeling efficiency and sequence-resolution, short non-repetitive genome segments such as *cis*-regulatory elements (enhancers and promoters) whose size is normally only approximately a few kb, can be effectively visualized. The MB-FISH method can also be utilized to visualize multiple loop interactions and delineate the pairwise interactions in single cells with high resolution.

Cremer et al. utilized probes generated from conventional nick translation PCR to target a 6-kb-long locus and obtained images using 3D structured-illumination microscopy (3D-SIM) (Cremer et al. 2017). Not as sophisticated a probe design and production procedure as “Oligopaint” or “MB-FISH,” this method used two differently labeled probe sets to label two 6-kb-long adjacent loci simultaneously to identify true hybridization events based on co-existing signals from both probe sets. Because two sets of differently labeled probes are needed to reliably target a small genomic region at one time, this method might be less versatile in co-labeling of multiple targets considering the total available fluorescent dyes.

Despite the improvement in the FISH-probe design, the choice and chemistry of fluorophores used in FISH experiments are also important concerns (Dempsey et al. 2011). Nanotechnology holds great potential for the study of genome structure. Semiconductor nanocrystals, i.e., quantum dots (QD), were quickly adopted into biological labeling due to their superior fluorescence properties after synthesis (Murray et al. 1993). Pathak et al. demonstrated the first CdSe/ZnS (core/shell) QD-ISH to the Y chromosome in fixed cells (Pathak et al. 2001), indicating that stable aqueous mono-dispersity and minimized non-specific labeling are crucial for a successful QD-ISH. Direct-labeled QD-ISH probes have been reported to image repetitive sequences and specific genomic loci (Ma et al. 2008). However, these direct-labeled QD probes usually require chemical synthesis, which limits their adaptation. The commercialized QD-conjugated antibodies against haptens (for example, digoxigenin, biotin) enable one to develop indirect QD-ISH methods similar to FISH-IF (immunofluorescence), which can be applied in biology and clinical diagnosis. Xiao et al. made the first demonstration of this strategy by labeling both repetitive sequences and specific gene locations on metaphase chromosomes (Xiao and Barker 2004). Muller et al. expanded the QD-ISH with chromosome-painting probes to label the whole chromosome (Muller et al. 2009), similar to the chromosome territory FISH method (Bolzer et al. 2005). However, it remains challenging to fully potentiate nanoparticles (including QD, surface-enhanced Raman scattering nanoparticles, upconversion nanoparticles, and silica nanoparticles) for chromatin labeling due to their low reliability and consistency in quality control (Ioannou and Griffin 2010). The size and surface chemistry of each nanoparticle should be uniformly synthesized and well-adjusted to fit in a special biological sample. On the other hand, micro- and nano-devices have been successfully adopted to probe epigenomic states and chromatin interactions (Aguilar and Craighead 2013), as well as single-cell sequencing (Blainey and Quake 2014).

Despite all the contributions made by FISH-based methods in both DNA and RNA studies, the drawback of these methods is also obvious, especially in DNA labeling. Because FISH requires base-pairing between the target DNA and probes, a treatment of heat denaturation is needed to denature the native DNA double helix to allow access of the probes. Considerable effort has been made to preserve the nucleus morphology and chromatin ultra-structure during FISH procedures (Markaki et al. 2013). Oligopaint-FISH with the heat denaturation step provides loci-spatial organization results consistent with Hi-C data (Wang et al. 2016b). However, it should be noted that the DNA ultrastructure at the level of nano-resolution might still be distorted. Hence, the development of DNA labeling methods without the need for heat denaturation is still the focus of great anticipation.

Short peptide nucleic acid (PNA) probes have been used to label specific repetitive DNA sequences in situ (mostly telomere, centromere) without a heat denaturation step (Genet et al. 2013). Since PNA probes can bind to a complementary sequence of DNA with higher affinity than the DNA would have when binding with itself, PNA probes can gain sufficient access to the DNA targets even without denaturation of the original DNA double-helix. Unfortunately, considering the cost for the synthesis of PNA probes, people generally do not consider PNA a practical method. Therefore, there are no reports yet using PNA probe sets to label non-repetitive DNA loci in situ. However, it is theoretically possible to label specific genomic loci with PNA-FISH without heat denaturation.

### Imaging RNA in cell nuclei

Though the direct link between RNA species and the 3D genome architecture regulation is still being investigated, there is growing evidence that some RNA species, such as long noncoding RNA (lncRNA), can be important players in shaping the 3D genome (Davidovich and Cech 2015). lncRNAs, nascent RNAs, and small nucleolar RNAs (snoRNAs) can meditate regulatory activities on chromatin, and genomic RNA–chromatin interactions are directly linked to transcription. Many RNAs can reach out to chromatins which are Mb away in linear DNA distance, and some RNAs can even decorate an entire chromosome arm, indicating that there are general patterns of RNA–chromatin interactions. Therefore, developing novel imaging tools to image (and to track) individual chromatin-interacting RNAs will be helpful to understand how 3D genome organization helps gene regulation and transcription.

The first RNA FISH probes were modified nucleotides that incorporated antisense RNA generated from plasmid templates and were further targeted by antibodies that could be labeled and imaged (Langer et al. 1981). Later, sets of short synthetic DNA probes tagged with fluorophores were invented for better RNA FISH performance (Femino et al. 1998). By improving this oligo probe-based technique in terms of the size of the probe set and probe length, RNA FISH can be used to detect multiple RNA species in the same individual cells at the same time (Raj et al. 2008). Currently, this kind of RNA FISH method, with the use of single fluorophore-tagged short oligo probes, is so common and robust that many companies offer commercial probe synthesis for RNA imaging, such as Stellaris FISH (Orjalo Jr et al. 2011).

RNA-smFISH (RNA-single molecular FISH), which images single RNAs using transcript-specific probes, has been modified and advanced in terms of sensitivity and robustness (Itzkovitz and van Oudenaarden 2011). Most RNA-smFISH methods are manipulated in studying the transcriptome of individual cells.

Apart from using multiple linear singly labeled probes, other probe designs, such as molecular beacon (Tyagi and Kramer 1996) and branching DNA amplification (Player et al. 2001), are also available for the same aim of improving labeling signals and sensitivity. Based on these strategies, further refinements have been focused on multiplexing the RNA labeling capability and improving the RNA molecule imaging resolution, providing resourceful means for RNA studies (Lubeck and Cai 2012).

## Imaging 3D genomes in live cells

3D genome regulation is a dynamic process. Although FISH-based methods can capture a detailed chromatin architecture at a specific moment, they cannot monitor any potential dynamic changes. Therefore, a method to image specific genomic loci in live cells is indispensable. Fluorescent proteins fused with native DNA-binding proteins were first utilized to label DNA sequences, but the versatility and effectiveness of this approach were restricted to the limited choice of available DNA-binding proteins (Robinett et al. 1996).

**Fig. 2:**
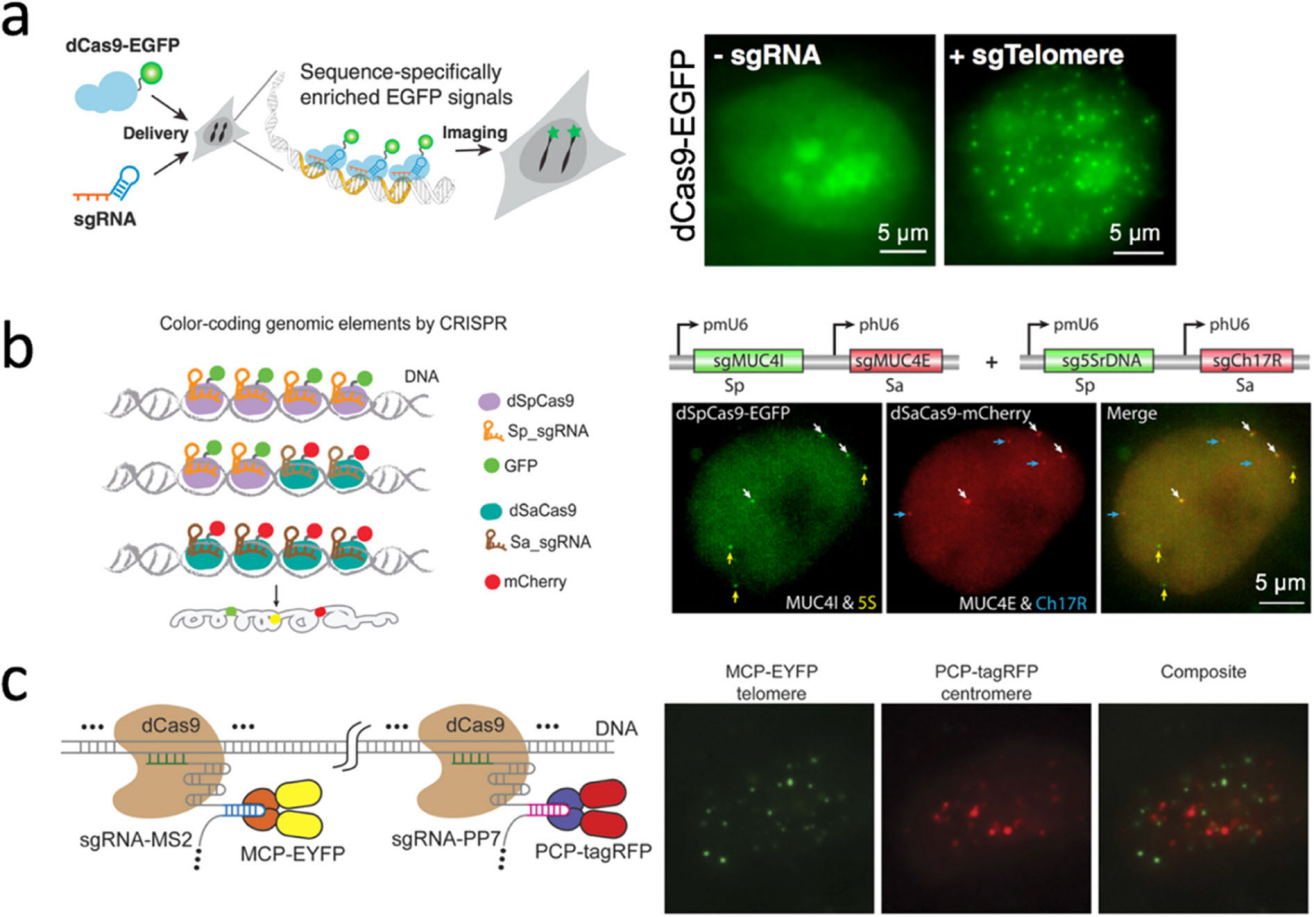
CRISPR-cas9 derived DNA labeling and imaging. **a** The system design (left) and image of labeling telomeres in live cells (right) (image modified from Chen et al. 2013). **b** The system design of orthogonal CRISPR/dCas9 systems from two bacterial sources allowed labeling genomic loci with multi-color (left). In an illustrative image of such a system, three loci (MUC4, 5S rDNA, and Ch17R) from chromosomes 1, 3, and 17, were simultaneously labeled, respectively, without interference (right) (image modified from Chen et al. 2016). **c** The system design of the CRISPR/dCas9 system utilizing MS2-MCP and PP7-PCP RNA aptamer-protein interaction pair (left) to allow labeling two different DNA loci with dual-color (right) (image modified from Wang et al. 2016a)

The type II CRISPR (clustered regularly interspaced palindromic repeats) system derived from *Streptococcus pyogenes* (Wiedenheft et al. 2012) provided a powerful platform that could recognize target DNA sequences with the Cas9 protein, whose binding specificity was solely determined by a small guide (sg)RNA and a protospacer adjacent motif (PAM) (Jinek et al. 2012). Cas9 protein, with its innate endonuclease activity, revolutionized genome editing using the CRISPR system, which is also notable in the field of bioimaging. By using the nuclease-deactivated version of Cas9 protein (dCas9) fused with fluorescent proteins and custom-synthesized sgRNA, the CRISPR/dCas9 system enabled imaging of specific DNA loci in live cells (Fig. 2a) (Chen et al. 2013).

Accompanied with the expanding discovery of Cas9 orthologs with distinct DNA binding specificity and PAM recognition from different species, it is possible to develop a multi-color imaging system by using different fluorescent proteins (Ma et al. 2015). For example, SpCas9 and SaCas9, Cas9 proteins that originated from the bacteria *Streptococcus pyogenes* and *Staphylococcus aureus*, respectively, are orthogonal in the same cell, can identify their own sgRNAs without affecting each other and could be utilized to detect different genomic loci at the same time (Fig. 2b) (Chen et al. 2016).

Instead of fusing a fluorescent protein to dCas9 orthologs, the CRISPR imaging system can be multiplexed by extending the sgRNA to include specific RNA scaffolding, namely, an RNA aptamer hairpin (Zalatan et al. 2015). This RNA aptamer, such as MS2 and PP7, could recruit specific proteins MCP and PCP, respectively, without interference. When fusing fluorescent proteins to each type of recruiting proteins, one can utilize various RNA aptamer-protein interaction pairs, such as MS2-MCP and PP7-PCP, to recognize different loci with different fluorescent colors (Fig. 2c) (Shao et al. 2016; Wang et al. 2016a). Since it is shown that the RNA-aptamer and protein pairs are also orthogonal to each other, different sources of CRISPR/dCas9 system and RNA-protein interaction pairs could be combined to potentiate the power of the CRISPR/dCas9 labeling system.

### Novel super-resolution microscopy (SDOM and low-power STED)

Different optical super-resolution microscopy techniques have been used to study the structure and dynamics of chromatins, and the application of these techniques to genome 3D structure has been reviewed intensively somewhere else (Gao et al. 2016). In this section, we will focus on only two novel super-resolution microscopy (SRMs) developed recently by us: (1) super-resolution dipole orientation mapping (SDOM) microscopy, which can detect the orientation of fluorescent dyes aligned to the DNA axis at specific degree and (2) low-power super-resolution STED nanoscopy.

### Dyes aligned to DNA axis

**Fig. 3:**
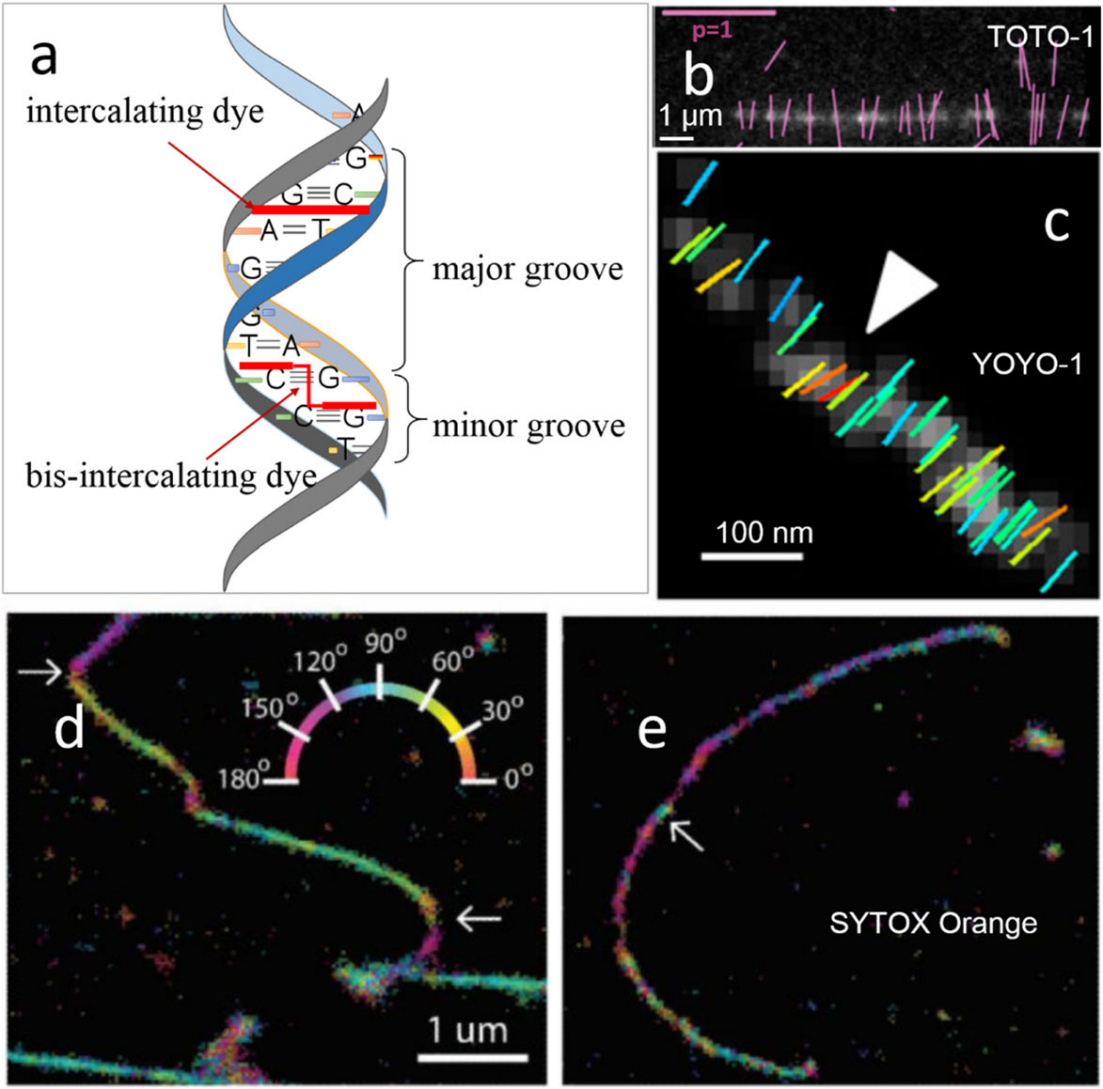
Imaging DNA in vitro using bis-intercalating or intercalating dyes. **a** A schematic diagram to show the binding modes of bis-intercalator and intercalator. **b** Fluorescence image of λ phage DNA stained with 50 nM TOTO-1 with orientations of polarized fluorescence of detected particles shown by magenta lines. Image modified from Mehta et al. 2016. (Scale bars: magenta, polarization factor = 1; white, 1 μm). **c** The arrowhead points to a location of the double stranded DNA fiber whose molecular order departs from the average behavior of the imaged region (images b and c modified from Valades Cruz et al. 2016). (Scale bar 100 nm). **d** Super-resolution image of a λ-DNA strand containing multiple “bends” (see arrows). **e** Super-resolution image of a λ-DNA strand exhibiting “tangles” (see arrow) (images d and e modified from Backer et al. 2016). (Scale bar 1um)

Bis-intercalating dyes, such as TOTO-1 and YOYO-1, firmly align perpendicularly to the DNA axis. The planar structure of these dyes has two conjugate moieties sandwiched in between consecutive base pairs (Fig. 3a) (Larsson et al. 1994), generally orienting perpendicular to the DNA axis. With the orientation information and absorption dipole moment obtained from fluorescent dyes inserted into or attached to DNA directly, we can detect DNA conformation changes and probe the structure of individual DNA strands.

Single-molecule orientation measurement can be combined with localization data to detect deformation that has been induced within DNA strands. Figure 3b presents the orientation of individual TOTO-1 fluorophores that were intercalated into combed DNA and had fluorescence that is polarized perpendicular to the DNA axis (Mehta et al. 2016). Cruza et al. observed locally double-stranded DNA fiber regions where the orientations of YOYO-1 do not follow the fiber average direction, indicating local bending (Fig. 3c) in the DNA filament (Valades Cruz et al. 2016).

SYTOX Orange, an intercalating dye (Fig. 3a), is expected to fit between adjacent base pairs, orienting approximately perpendicular to the DNA axis (Yan et al. 2000). Dye-DNA interactions were characterized by orientation analysis; for example, intercalating and groove-binding dyes show homogeneous and heterogeneous molecular order, respectively. DNA conformational changes could also be monitored using the intercalating dye SYTOX Orange (Backer et al. 2016). Moerner and his colleagues undertake exquisite analysis to resolve the spatial position, in-plane molecular orientation, and rotational immobility of the marker molecule. In Fig. 3d, single-molecule positions and orientations demonstrate that when the DNA axis abruptly bends, labeling density drops (see arrows). Figure 3e shows a patch of DNA-tangle (see arrow) where the dye molecule orientation varies wildly, followed by a neighboring strip along which the density of detected molecules declines (Backer et al. 2016).

### Polarized super-resolution microscopy (PSRM)

Polarization properties of the dye inserted into DNA and restricted orientation relative to the targeted DNA strand can be investigated with an imaging tool called polarized fluorescence microscopy, such as instantaneous FluoPolScope (Mehta et al. 2016). The method involves modulating the polarization of an excitation laser and analyzing the corresponding intensities emitted by dye molecules and their modulation amplitudes to image the position and orientation of fluorophores. Fluorescently labeled DNA in vitro can be tested by the instantaneous FluoPolScope system, in which the ensemble orientation of polarized fluorescence is known.

Recently, PSRM (Zhanghao et al. 2017) provided a novel means to probe orientation information on the local structure of DNA bases and to reveal information about the underlying DNA. Polarized direct stochastic optical reconstruction microscopy (polar-dSTORM) is reported to quantitatively analyze orientational order in filaments at the nanoscale (Valades Cruz et al. 2016). In polar-dSTORM, the signal was experimentally divided into two polarization states, and both localization and steady-state in-plane orientation of single-molecules with high precision can be analytically resolved, which results in super-resolution of polarized fluorescence and can be applied to morphological investigations such as protein–DNA interaction studies.

Karl Zhanghao et al. developed a new PSRM called SDOM microscopy to determine the dipole orientation of fluorescent molecules at the sub-second scale in live cells (Zhanghao et al. 2016). SDOM is built on wide-field epi-fluorescence illumination microscopy, and then polarization modulation can be implemented by using a rotary half-wave plate. In the presence of polarized laser light, the fluorescent molecules with inherent dipole orientations emit periodic signals, which are collected by a camera such as an electron-multiplying CCD. The fluorescent dipole orientation reflects the direction of the targeted protein (for example, transcription factors that bind to chromatin) as the fluorophore holds a preferential alignment along the target molecule. To analyze the polarized modulation of fluorescence images from a wide-field epi-fluorescence illumination, SDOM establishes a polarization-variant model to resolve the super-resolution microscopic images using sparse deconvolution first and then retrieves the phase of each super-resolved focal pixel using a least squares estimation. The dipole orientations are superimposed onto the image as arrows, whose direction and length denote effective dipole orientation and orientation uniformity accordingly. Due to high temporal resolution, SDOM has the potential to image the dynamics of DNA molecules or chromatin structures at the sub-second scale or even at a milli-second level (data not published yet).

### Low-power super-resolution STED microscopy

Traditional STED nanoscopy requires a high-power beam and thus an extremely high depletion intensity, which triggers severe photobleaching of fluorophores and damages biological samples; this forestalls the application of STED in many biology studies, especially time-lapse live imaging of genome structures (Liu et al. 2017).

Low-power super-resolution STED microscopy uses a low-cost, low-power, near-infrared diode laser (7-mW 808-nm laser) for excitation of upconversion nanoparticles (UCNPs), which reduces the depletion power by two orders of magnitude, to achieve 28-nm optical resolution (λ/36) for imaging UCNPs. Though it is not yet used to image 3D genome structures because it is still hard to reduce the non-specific binding of UCNPs during ISH experiments, this technique holds great potential for time-lapse super-resolution live-cell imaging of chromatin, especially in deep tissue (Liu et al. 2017).

## Visualizing 3D genomes

Biochemical and microscopic techniques generate first-hand data to understand 3D genomes from a specific angle, depending on the experimental approach. Taken another step further, the data generated can also be used for computational analysis on structural prediction and visualization, which is essential in bioinformatics and structural biology to deepen the understanding of biomolecules, including proteins and nucleic acids.

Owing to the rendering efficiency bottleneck of 3D computer graphics, traditional molecule structure viewers are often limited to the native desktop environment (O'Donoghue et al. 2010; Pirhadi et al. 2016). PyMOL (http://www.pymol.org/) is a commonly used software for molecular visualization because of its high rendering efficiency and fine graphical quality. With the development of Internet technology, there is a widespread need for visualization tools to present the 3D structures of biomolecules in web browsers. Jmol (http://sourceforge.net/projects/jmol/) (Hanson 2010) and OpenAstexViewer (http://www.openastexviewer.net/) (Hartshorn 2002) are representative web-based viewers implemented as Java Applets, which means that a Java runtime plugin must be preinstalled into every web browser accessing Jmol or OpenAstexViewer, and a series of security options must be found and checked off before the programs can finally run.

**Fig. 4:**
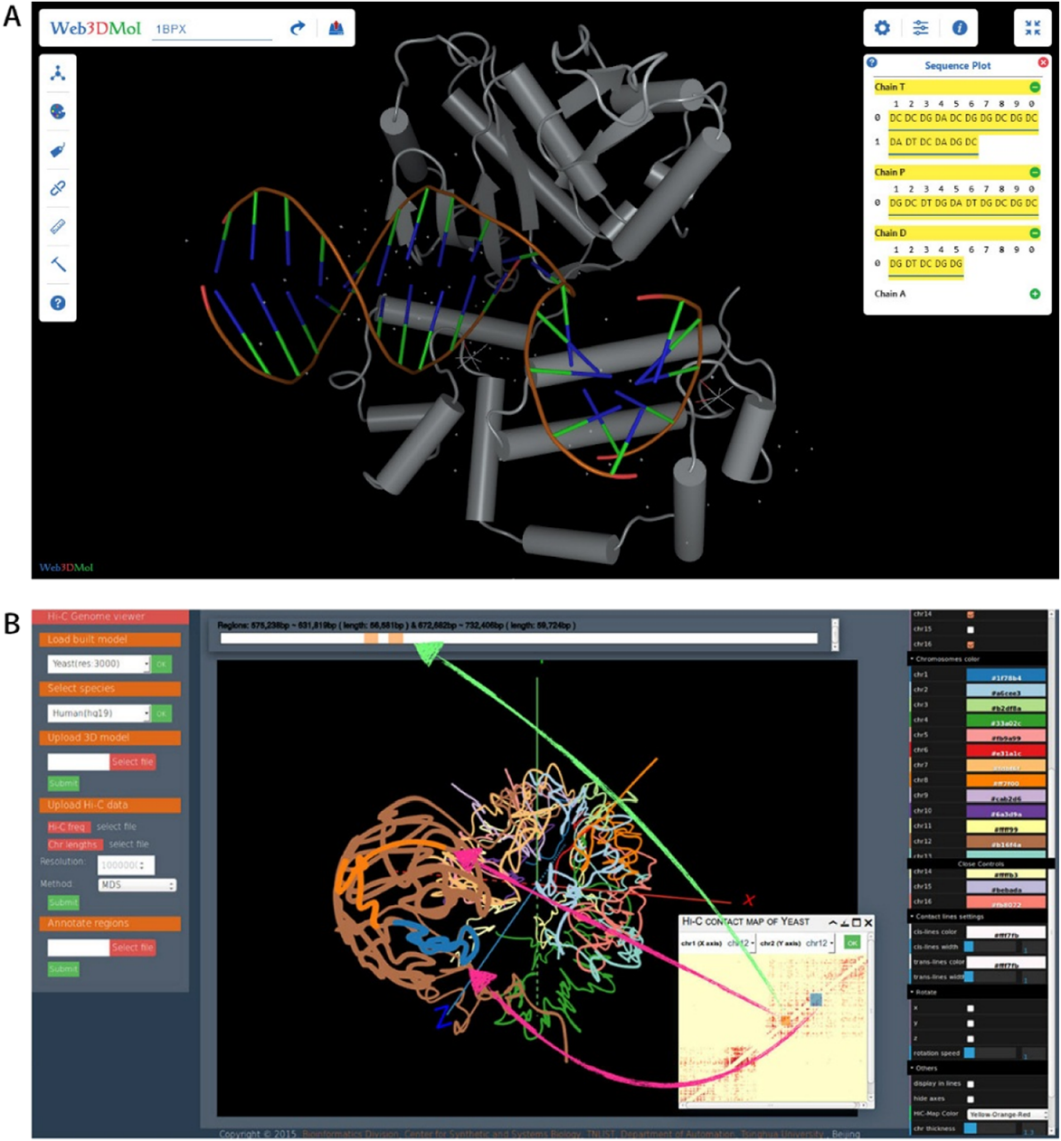
Visualization tools developed recently to visualize proteins and genome in 3D space. **a** A DNA-protein complex (PDB-ID: 1BPX) is shown in Web3DMol. Schematic diagram produced by the software in Shi et al. 2017. **b** HiC-3DViewer can highlight genomic regions of interest in a Hi-C frequency matrix interactively and visualize them in 3D space with 1D-to-2D-to-3D mapping (image modified from Djekidel et al. 2016).

With the popularity of HTML5, WebGL (http://www.khronos.org/webgl/) has been widely accepted and supported by web browser manufacturers as the standard 3D API. A growing number of web-based databases for nucleic acid and protein research are being developed. NGL (http://github.com/arose/ngl) (Rose and Hildebrand 2015) and Web3DMol (http://web3dmol.duapp.com/) (Shi et al. 2017) are representative web-based molecule visualization tools using WebGL, which provide interactive 3D presentations of molecule structures and allow users without any programming experience to construct desirable graphics. Figure 4a shows the visual effect of Web3DMol.

In addition to biomolecule viewers, other tools are also available for specific genome structure visualization. HiC-3DViewer (Djekidel et al. 2016) is a browser-based interactive tool designed to provide an intuitive environment to facilitate the 3D exploratory analysis of Hi-C data (Fig. 4b). The 1D-to-2D-to-3D mapping between nucleic acid sequences, Hi-C matrix and genome 3D structures can be inspected by users with HiC-3DViewer. Because of the complexity of genomes, it is difficult to visualize entire genomes and the detailed structures at the same scale. GMOL (Nowotny et al. 2016) is a useful tool which allows users to scale between six separate levels to inspect the spatial structure of the genome and retrieve corresponding genome sequences.

## Conclusion

In this review, we introduced two of the three strategies (proximity ligation-based methods, imaging tools, and computational/visualization methods) used frequently to study 3D genome structures, namely imaging and visualization methods. Different methods to image DNA and RNA in fixed or live cells were introduced, the pros and cons were compared (see Table 1), and the methods that do not use heat denaturation were introduced thereafter. It should be noted that labeling and imaging of RNA in individual cell nuclei is also important for 3D genome study, as many RNAs could bind to chromatins, which are Mb away in linear DNA distance. Direct co-imaging of the RNA target and chromatin landscape may give us more valuable knowledge of the how RNA could modify the topology of chromatin.

**Table 1:** The comparison of different DNA imaging methods

	FISH	MB-FISH	CRISPR/dCas9 system
Resolution	100–200 kb	2.5 kb	5 kb
Time	~ 2 days	~ 1 month	~ 1 week
Cost (US dollars)	~ 225	~ 12,000	~ 600
Sample type	fixed cell	fixed cell	live cell/fixed cell

The photon-capturing capability of optical microscopes is very important for the sensitivity of detecting DNA/RNA fluorescence signals, so we next introduced two super-resolution microscopy methods, SDOM, which is available for detecting dipole orientation and dynamics of proteins in live/fixed cells (for example, neurons, kidney slice), and low-power STED, which holds great potential for time-lapse super-resolution live-cell imaging of chromatins or 3D genomes, especially in deep tissue. A comparison of different SRM methods is presented in Table 2.

**Table 2:** The comparison of different super-resolution microscopy methods

Properties	SPoD	SDOM	Low-power STED	Polar-dSTORM
Spatial resolution	~ 90 nm	~ 90 nm	~ 30 nm	~ 20 nm
Temporal resolution	At sub-second scale	At sub-second scale	At second scale	At several minutes scale
Number of frames	~ 10	~ 10	NAN	~ 10^3^
Polarization that reveals rotary information along filaments	No	Yes	No	Yes
Excitation laser power	Several mW	Several mW	Several tens mW	Several mW

Structure visualization of biomolecules, including proteins and nucleic acids, is an essential task in bioinformatics and structural biology. Therefore, at the end of this article, we highlighted two browsers developed by our group after discussing the pros and cons of visualization tools developed by others.

Though it is still difficult to image specific genome loci in live cells because of the challenge of specificity and low signal-to-noise ratio, with the growing power of novel super-resolution microscopy, fluorescent probes, imaging analysis algorithms, and high-throughput proximity ligation-based techniques, we will be able to gain more insight into the fundamental principles behind the temporal and spatial regulation of transcription in 3D genomes.

Finally, for 3D genome organization studies, the three strategies introduced above are not mutually exclusive. Instead, these three methods are complementary to each other, and the combination of the three main methods to study 3D genomes can help us better understand how chromatin folding and unfolding in 3D space contribute to the regulation of genes. For example, in Hi-C, ChIA-PET, GRID-seq and other 3C-based experiments, intrachromosomal or interchromosomal interactions were detected based on statistical models and algorithms (Li et al. 2017), and FISH was employed intensively to confirm the interactions between chromatin loci (Fullwood et al. 2009). These experiments indicate that these three strategies are complementary to each other and should not be employed separately. In the 3D FISH experiment, the surface construction of target gene loci, chromosomes, and cell nuclei through segmentation, surface rendering, and other imaging process algorithms (Bolzer et al. 2005; Murmann et al. 2005) can help us better understand how chromatins are organized in 3D space. Though there could be some disagreement among these three strategies at specific regions (Yu and Ren 2017), the integration of these three methods is the main trend for people to better explore 3D genomes in different species (Knoch et al. 2016; Wachsmuth et al. 2016).
